# Astrocyte Glutamate Uptake and Signaling as Novel Targets for Antiepileptogenic Therapy

**DOI:** 10.3389/fneur.2020.01006

**Published:** 2020-09-08

**Authors:** Allison R. Peterson, Devin K. Binder

**Affiliations:** Division of Biomedical Sciences, Center for Glial-Neuronal Interactions, School of Medicine, University of California, Riverside, Riverside, CA, United States

**Keywords:** epilepsy, astrocytes, glutamate transporters, metabotropic glutamate receptors, GLT-1, GLAST, mGluR3, mGluR5

## Abstract

Astrocytes regulate and respond to extracellular glutamate levels in the central nervous system (CNS) *via* the Na^+^-dependent glutamate transporters glutamate transporter-1 (GLT-1) and glutamate aspartate transporter (GLAST) and the metabotropic glutamate receptors (mGluR) 3 and mGluR5. Both impaired astrocytic glutamate clearance and changes in metabotropic glutamate signaling could contribute to the development of epilepsy. Dysregulation of astrocytic glutamate transporters, GLT-1 and GLAST, is a common finding across patients and preclinical seizure models. Astrocytic metabotropic glutamate receptors, particularly mGluR5, have been shown to be dysregulated in both humans and animal models of temporal lobe epilepsy (TLE). In this review, we synthesize the available evidence regarding astrocytic glutamate homeostasis and astrocytic mGluRs in the development of epilepsy. Modulation of astrocyte glutamate uptake and/or mGluR activation could lead to novel glial therapeutics for epilepsy.

## Introduction

Epilepsy is a common neurological disorder and is characterized by the occurrence of unprovoked seizures. Epilepsy is a major public health problem, affecting more than 65 million people worldwide (1). Healthcare cost estimates associated with epilepsy in the United States range from $9.6 to $12 billion per year (2). TLE is the most common form of epilepsy with focal seizures. TLE is also frequently associated with refractory epilepsy. Approximately 1/4 of patients with TLE develop refractory epilepsies that are pharmaco-resistant to currently available antiepileptic drugs (AEDs) (3).

AEDs work primarily by targeting neurons through modulation of ion channels, enhancement of inhibitory neurotransmission or attenuation of excitatory neurotransmission (4). Most AEDs target channels on neurons to exert their antiepileptic effects. Newer generation AEDs still primarily target neurons but through novel mechanisms and unique binding sites [e.g., AMPA-R, CMRP2, SV2A, or inhibition of carbonic anhydrase activity (5)]. Modulation of neurotransmission can consequently lead to dose-dependent “neurotoxic” adverse effects which are common undesired effects associated with AED usage. Adverse cognitive and behavioral effects of AEDs have been shown to lead to AED discontinuation in up to one-third of patients (6). Therefore, new non-neuronal targets that could potentially have fewer side effects should be considered and further investigated.

Neuronal hyperexcitability is a major contributor to epilepsy but increased evidence suggests that changes in astrocytes can contribute to the development of epilepsy (7–13). Astrocytes are involved in ionic homeostasis, regulation of extracellular space volume and clearance of neurotransmitters. Astrocytes are a critical component of the tripartite synapse, where they are involved in the active control of neuronal activity and synaptic neurotransmission. Astrocytes regulate extracellular glutamate levels *via* Na^+^-dependent glutamate transporters, glutamate transporter-1 (GLT-1) and glutamate aspartate transporter (GLAST). GLT-1 is responsible for ~90% of glutamate uptake in the adult dorsal forebrain and is crucial for the maintenance of low extracellular glutamate to permit efficient synaptic transmission (14). The human homologs of GLAST and GLT-1 are EAAT1 and EAAT2, respectively. In this review we will be referring to these transporters in pre-clinical and clinical studies by GLAST and GLT-1. Aside from perisynaptic glutamate uptake, astrocytes can also sense extracellular glutamate to more readily adapt to their microenvironments through metabotropic glutamate receptors mGluR3 and mGluR5. These G-protein coupled receptors can differentially modulate the expression of glutamate transporters and glutamate release therefore indirectly regulating synaptic activity. This review will provide an overview of what we currently understand regarding the regulation of astrocytic glutamate transporters and receptors in the development of epilepsy. Targeting glutamate uptake and/or glutamate receptor activation through astrocytes could lead to novel treatment options for patients with refractory epilepsies.

## Dysregulation of Glutamate Uptake in Epileptogenesis

**Figure 1:**
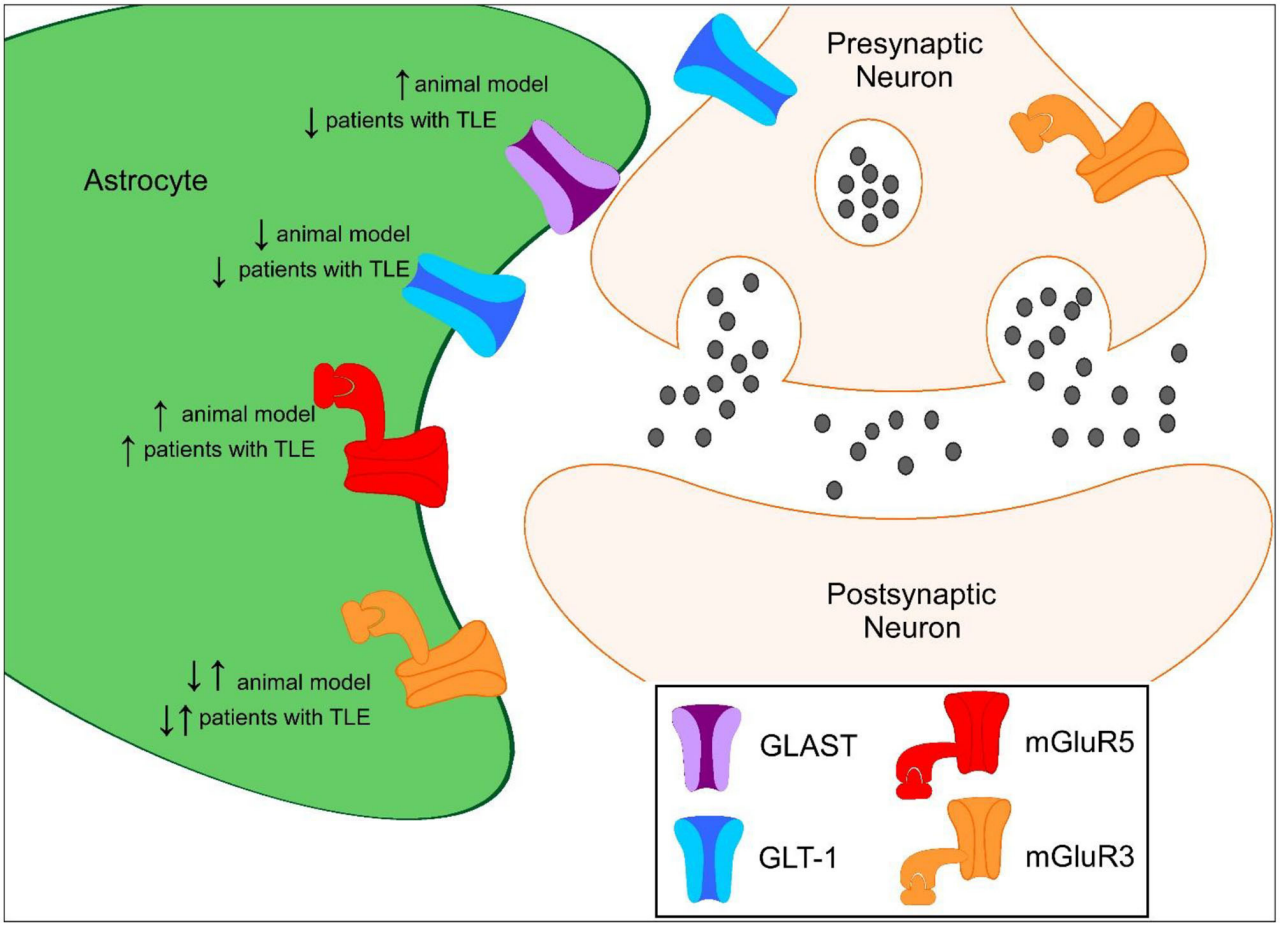
Proposed protein expression of glutamate transporters and mGluRs in the hippocampus during epileptogenesis compared to controls. ↑ represents increased protein expression observed compared to control. ↓ represents decreased protein expression observed compared to control. ↓↑ represents decreased and increased in protein expression observed compared to control.

GLT-1 and GLAST are the primary transporters responsible for glutamate clearance in the central nervous system (CNS) following excitatory neuronal transmission. It is crucial to maintain low levels of basal extracellular glutamate in the brain to permit efficient and localized synaptic transmission. Evidence of increased glutamate levels have been observed in patients suffering from TLE and in preclinical seizure models (15–17). The vast majority of GLT-1 is astrocytic with synaptic localization, with ~5–10% of expression in neurons (18, 19). Mice that globally lack GLT-1 develop lethal spontaneous seizures, while transgenic mice that overexpress GLT-1 have a higher seizure threshold than wild-type mice, suggesting that GLT-1 plays an important role in preventing seizures and protection against glutamate toxicity (20, 21). In multiple preclinical studies, GLT-1 protein levels have been shown to be downregulated during the development of epilepsy (Figure 1). Perisynaptic GLT-1 at the plasma membrane in astrocytes is significantly reduced around CA3-CA1 synapses during the latent period following systemic kainate-induced status epilepticus (SE) (22). Hippocampal GLT-1 total protein levels have been found to be downregulated following intrahippocampal kainate-induced SE (11). Crude synaptosomal GLT-1 levels, which include components of the tripartite synapse, are also reduced nearly 80% 1 week following intrahippocampal kainate induced-SE in the hippocampus early in the epileptogenic process (13). These data suggest that the pool of transporters available for glutamate uptake at excitatory synapses is substantially reduced in epileptogenesis. The kainic acid (KA) model of TLE is characterized by a period of SE, that serves as the initial insult, followed by a latency period where the mice are seizure-free followed by the occurrence of spontaneous recurrent seizures (23–25). Downregulation in GLT-1 protein levels observed in these studies interestingly coincides with the approximate onset of spontaneous seizures, demonstrating that glutamate transporter dysregulation could contribute to the development of epilepsy (13, 22, 26–28). Interestingly, GLT-1 protein levels were found to be upregulated in in a spontaneously epileptic rat, a double mutant (zi/zi, tm, tm), compared to control Wistar rats (29). GLT-1 protein levels have also been shown to be disrupted in patients with TLE (8, 9). GLT-1 levels have been found to be decreased in the hippocampus of TLE patients with hippocampal sclerosis (HS) in most (8, 9) but not all (30) studies. In patients with decreased GLT-1, severe neuronal cell loss was observed suggesting that loss of glutamate transporters could exacerbate neurotoxicity in epilepsy (8, 9).

GLAST has also shown to be dysregulated in the epileptic brain. GLAST is found in the dorsal forebrain postnatally and homogenously distributed among astrocytic soma and endfeet compared to its counterpart GLT-1 (18, 31). GLAST-deficient mice have significantly longer seizure duration compared to wild-type mice suggesting that GLAST also plays a role in seizure susceptibility (32). In a preclinical model of TLE, synaptosomal GLAST protein levels were elevated in the epileptic hippocampus while overall total protein levels were unchanged at chronic time points (13). GLAST protein levels were found to be significantly lower in a spontaneously epileptic rat, a double mutant (zi/zi, tm, tm), compared to control Wistar rats (29). In TLE patients with HS, GLAST protein levels have been shown to be downregulated while GLAST protein levels are unchanged in TLE patients without HS (8). Astrocytic glutamate synthetase is responsible for the rapid conversion of intracellular glutamate to glutamine and is a prerequisite for efficient glutamate clearance from the extracellular space. Loss of glutamine synthetase has also been observed in patients with TLE which could have an impact on glutamate transporter clearance (30). These findings suggest that glutamate transporter dysregulation could contribute to increased extracellular glutamate and ictogenesis in the epileptic brain.

## Regulation of Astrocytic Glutamate Receptors in Epileptogenesis

Metabotropic glutamate receptors (mGluRs) are G-protein coupled receptors (GPCRs) important in synaptic neuromodulation. These receptors can be divided into three separate families: Group I, Group II, and Group III, based on their structure and downstream function (33). Metabotropic glutamate receptors found on astrocytes can influence astrocytic functions in physiology and disease. Astrocytes dominantly express mGluR3 and mGluR5 receptors and differential regulation of these receptors has been observed in epilepsy (10, 34, 35). Astrocytic mGluR5 signaling plays an important role in astrocytic motility, ensheathment and glutamate transport in the developing brain (36, 37). Expression of astrocytic mGluR5 is typically limited to the first few weeks of brain development (37–39). Activation of Group I mGluR5 receptors, coupled to Gα_q_ proteins, has been shown to acutely alter GLT-1 activity by increasing glutamate clearance in astrocytes (40) but chronic stimulation can lead to a reduction in astrocytic GLT-1 and GLAST levels resulting in reduced glutamate transport (35).

Differential expression of astrocytic mGluRs have been reported in patients and preclinical models of epilepsy. mGluR5 levels have been shown to be overexpressed in murine seizure models. mGluR5 expression in reactive astrocytes is persistently upregulated following electrically induced SE in a kindling model and in TLE (34). Selective positive modulation of mGluR5 in the Theiler's murine encephalomyelitis virus (TMEV)-induced model of epilepsy attenuates seizures (41). Additionally, selectively knocking out astrocytic mGluR5 signaling during epilepsy slows glutamate clearance through glutamate transporters, suggesting that mGluR5 plays an important function in regulating these transporters in epileptogenesis (42).

Multiple studies have shown that mGluR5 levels are also increased in patients with TLE (10, 43). mGluR5 expression levels in patients have been associated with seizure frequency. Lower mGluR5 expression was found to be negatively correlated with seizure frequency and epilepsy duration in patients with TLE (non-HS) (10). Conditional knockout of mGluR5 signaling from astrocytes slowed glutamate clearance in epileptogenesis (42). These data support the hypothesis that mGluR5 upregulation could act as a compensatory mechanism to counterbalance the hyperexcitability observed in epilepsy.

It is important to note that activation of mGluR5 has also been shown to lead to increased excitability. For example, stimulation of group I mGluRs, including mGluR5, elicits ictal-like events in hippocampal slices (44). Following SE, mGluR5 activation has also been shown to enhance astrocytic calcium signals during the latency period of epileptogenesis in the pilocarpine model of TLE (45). Moreover, increases in astrocytic calcium transients can lead to release of gliotransmitters, including glutamate, and activation of NMDA receptors (46). In one study, intrahippocampal perfusion of the mGluR group 1 agonist, DHPG (R,S-3,5-dihydroxyphenylglycine), induced seizures while infusion of the mGluR5 receptor antagonist, MPEP (2-methyl-6-(phenylethynyl)-pyridin), attenuated pilocarpine-induced seizures (Table 1) (52).

**Table 1:** Positive and negative outcomes of glutamate transporter modulation and mGluR agonists/antagonist in preclinical seizure models.

**Drug candidate**	**Selectivity**	**Dose**	**Model**	**Antiepileptic effect**	**Other effects**	**References**
17AAG	HSP90β inhibitor	50 μl, 200; mg/kg, i.p.	KA model of TLE	↓ seizures	↑ GLT-1 ↓ astrogliosis	(47)
Ceftriaxone	GLT-1 transcriptional activator	200 mg/kg; i.p.	Knock out mouse model of TSC	↓ seizures	↑ GLT-1 ↓ glutamate ↓ neuronal death	(48)
APDC	Group II mGluR agonist	0.6 nmol; i.c.v. infusion	DL-HCA model of seizure	↓ seizures		(49)
APDC	Group II mGluR agonist	12.5, 50, 200, 400, and 600 mg/kg; i.v.	Pilocarpine model of TLE	No effect		(50)
DCG-IV	Group II mGluR agonist	0.5 μl, 1 nm; intra-amygdaloid	Kindling of the basolateral amygdala	↓ seizures		(51)
DCG-IV	Group II mGluR agonist	0.6 nmol/side; i.c.v. infusion	DL-HCA model of seizure	Partial effect		(49)
DCG-IV	Group II mGluR agonist	5–100 nmol/side; i.c.v. infusion	DL-HCA model of seizure	↑ seizures		(49)
DCG-IV	Group II mGluR agonist	1 μM; intrahippocampal	Pilocarpine model of TLE	Partial effect	↓ extracellular glutamate	(52)
DCG-IV	Group II mGluR agonist	10 μ; intrahippocampal	Pilocarpine model of TLE	Partial effect	↑ glutamate and GABA	(52)
DHPG	Group I mGluR agonist	1 mM; intrahippocampal	Pilocarpine model of TLE	↑ seizures	↑ glutamate and GABA	(52)
Cyclobutylene AP5	Group II mGluR agonist	4, 8, and 16 nmol/side; i.c.v. infusion	DL-HCA model of seizure	↓ seizures		(49)
MPEP	mGluR5 antagonist	50 mg/kg; i.p.	Pilocarpine model of TLE	↓ seizures	↓ glutamate and GABA	(52)
MPEP	mGluR5 antagonist	1 μg/g; I.V.	Pilocarpine model of TLE	No effect	↓ neuronal death	(45)

*Up-arrow notation represents an increase and down-arrow notation represents a decrease in the table*.

Hyperexcitability associated with mGluR5 activation has also been observed in other neurological diseases. For example, preclinical data suggest that in Fragile X Syndrome, a genetic form of autism, the absence of fragile X mental retardation protein (FMRP) leads to overstimulation of the mGluR5 pathway enhancing glutamatergic signaling contributing to phenotypes observed in this disease (53–55). Interestingly, treatment of *Fmr1* knockout mice with negative modulators of mGluR5 ameliorates phenotypes (56, 57). These studies indicate that although acute activation of mGluR5 can decrease excitability, chronic stimulation, which could occur in a diseased state, can be detrimental.

Activation of Group II mGluR3 receptors, which are coupled to Gα_i_ proteins in astrocytes, may have neuroprotective functions including increasing the capacity for glutamate clearance in the CNS through upregulation of glutamate transporters (35, 58). mGluR3 receptor activation has been shown to upregulate GLT-1 and GLAST protein levels promoting increased glutamate uptake in astrocytes (35, 58). mGluR3 receptors are also found in the presynaptic terminals of glutamatergic neurons (59). mGluR3 receptor agonists have also been shown to protect neurons from excitotoxicity and astrocytes from nitric oxide-induced death (60). Astrocyte-specific mGluR3 expression is markedly increased at early and chronic time points following SE in CA3 and hilar region following electrically induced SE in a kindling model and in TLE (34). A reduction in astrocyte-specific mGluR3 was observed in the molecular layer and stratum lacunosum moleculare of the hippocampus at chronic time points (34). mGluR2/3 expression was also found to be markedly decreased both acute and chronic time points following pilocarpine-induced SE (50).

Whether mGluR3 expression levels are upregulated or downregulated in patients with TLE is controversial. One study found mGluR2/3 expression is downregulated (50) while a separate study showed that mGluR2/3 is upregulated in the hippocampi of TLE patients (61). Whether this discrepancy is due to study design, severity or stage of epilepsy, region-specific effects, or technical differences remains to be determined. Future studies could further examine the use of selective negative modulators of mGluR5 or positive modulators of mGluR3 as an alternative therapeutic approach to treat epilepsy.

## Astrocytic Glutamate Uptake and Targeting of Glutamate Receptors as Therapies for Refractory Epilepsies

Non-neuronal targets, including glial cells, are an attractive alternative approach to treat patients whose seizures are not well-controlled with currently available AEDs. Increasing astrocytic glutamate uptake capacity by upregulation of glutamate transporters has been shown to have neuroprotective and anti-epileptic effects. Seizures were significantly reduced and astrogliosis was attenuated when mice were administered an HSP90β inhibitor to increase GLT-1 expression in a mouse model of TLE (47). Ceftriaxone, a β-lactam antibiotic, has also been shown to upregulate GLT-1 protein expression and reduce seizures in multiple preclinical studies (48, 62, 63). Treatment with ceftriaxone has shown negative adverse side effects including impairment in synaptic plasticity and memory recognition (64, 65). Ceftriaxone affects many pathways in the CNS, therefore, it is currently not well-understood if these adverse effects are a result of GLT-1 activation. Nevertheless, selectively targeting aberrant astrocytes could reduce adverse side effects. Intraspinal delivery of AAV8-Gfa2-GLT1 has been used to selectively increase GLT-1 protein expression under the truncated glial fibrillary acidic protein promotor in a model of spinal cord injury showing promising results (66). Gene therapy could potentially be used to target subpopulations of astrocytes by selecting genes known to be overexpressed in the epileptic brain. For example, adenosine kinase is strikingly upregulated in reactive astrocytes after kainic acid-induced SE and its promotor could be used to selectively target this cell population (67).

The mGluR5 receptor antagonist MPEP reduced seizures when administered i.p. in the pilocarpine seizure model (52). In contrast, another study also using the pilocarpine seizure model found that MPEP suppressed neuronal death but did not result in a change in synaptic activity, suggesting that astrocytes could have neurotoxic roles in epilepsy through increased gliotransmission (45). Future studies should further examine mGluR5 antagonists as potential adjunctive therapies to decrease the severe neuronal loss observed in TLE patients with HS. The mGluR2/3 agonist, APDC, was shown to reduce seizure in the DL-homocysteic acid (DL-HCA) seizure model (49). In the pilocarpine seizure model, APDC did not reduce seizures nor neuronal death (50). These studies indicate that selection of agonist/antagonists can have differential outcomes. Two Group II mGluR agonists targeting mGluR2/3, cyclobutylene AP5 and DCG-IV, have both demonstrated positive effects on seizure control in the DL-homocysteic acid (DL-HCA) seizure model and kindling model of TLE (49, 51). Interestingly, at higher doses DL-HCA has been shown to have pro-epileptic effects (49, 52). Thus, activation of Group II mGluRs may be another promising avenue for alternative therapies for treating epilepsy.

## Conclusion

Astrocytes play a critical role in the development and progression of epilepsy (7, 8, 30, 68–76). Astrocytic glutamate uptake is dysregulated in both preclinical models and in patients with TLE leading to increases in basal glutamate levels, and activation and signaling of astrocytic metabotropic glutamate receptors, mGluR3 and mGluR5, is also altered in animal models and patients with TLE. It is not clear yet whether targeting glutamate transporters and receptors would be more effective as a novel antiepileptic (controlling seizures in pharmacoresistant epilepsies) or antiepileptogenic (disease-modifying prevention of development of epilepsy after epileptogenic insults) strategy. Future studies should distinguish antiepileptic vs. antiepileptogenic effects, for example of GLT1 upregulation in appropriate animal models. Targeting of altered “epileptic” glutamate metabolism and signaling in astrocytes has the potential of efficacy with fewer side effects compared to traditional suppression of glutamatergic neurotransmission in neurons. This could lead to novel approaches to antiepileptic, antiepileptogenic, and/or neuroprotective therapies.

## Author Contributions

AP did an exhaustive literature search, generated a complete draft of the review, and prepared the table and figure. DB also reviewed the literature, provided detailed comments, and edits to the review and the table and figure. All authors contributed to the article and approved the submitted version.

## Conflict of Interest

The authors declare that the research was conducted in the absence of any commercial or financial relationships that could be construed as a potential conflict of interest.
